# Supplementary material for: Between synchrony and turbulence: intricate hierarchies of coexistence patterns
Source: Nat Commun. 2021 Sep 24;12:5634. doi: 10.1038/s41467-021-25907-7 (PMC8463560; doi:10.1038/s41467-021-25907-7)
Supplement: Supplementary file 1 — Supplementary Information [file 41467_2021_25907_MOESM1_ESM.pdf]

Supplementary Information: Between Synchrony and Turbulence: Intricate Hierarchies of Coexistence Patterns

Sindre W. Haugland, Anton Tosolini, and Katharina Krischer[a)]

Physics Department, Nonequilibrium Chemical Physics, Technical University of Munich, James-Franck-Str. 1, D-85748 Garching, Germany

(Dated: 23 August 2021)

The following Supplementary Figures as well as the sections describing the reasoning behind them are presented in the order the main manuscript addresses these figures.

SUPPLEMENTARY NOTE 1: c_2-INCREMENTED SIMULATIONS BEHIND THE SCHEMATICS IN FIG. 3 B-D

Like the schematic in Fig. 3 b is based on the quantitative simulation in Fig. 3 a, the schematic in Fig. 3 c is based on the quantitative simulation in Supplementary Fig. 1, and the schematic in Fig. 3 d is based on the quantitative simulations in Supplementary Figs. 2 and 3.

In Supplementary Fig. 1, the $N = 32$ ensemble is initialized in a $16-16$ configuration at $c_2 = -0.74$, $\nu = 0.1$ and $\eta = 0.63$. This corresponds to the very left of the figure, from where c_2 was incremented by $\Delta c_2 = 2 \cdot 10^{-5}$ every $\Delta T = 10^4$ time steps. The upper part of the figure depicts how the maxima of the real part of each oscillator evolve in the co-rotating frame of the ensemble average $\langle W \rangle = \eta \, \mathrm{e}^{-i\nu t}$. The lower part depicts the sizes of clusters automatically detected at each c_2 step. Further details are given in the figure caption.

In Supplementary Fig. 2, the $N = 256$ ensemble is initialized in a 128–64–64 configuration at $c_2 = -0.7145$, $\nu = 0.1$ and $\eta = 0.63$. This corresponds to the very left of the figure, from where c_2 was incremented by $\Delta c_2 = 10^{-5}$ every $\Delta T = 2 \cdot 10^4$ time steps. Again, the upper part of the figure depicts how the maxima of the real part of each oscillator evolve in the co-rotating frame of the ensemble average $\langle W \rangle = \eta \, \mathrm{e}^{-i\nu t}$. The lower part depicts the sizes of clusters automatically detected at each c_2 step. Further details are given in the figure caption.

Supplementary Fig. 3 depicts a continuation of the c_2-incremented simulation in Supplementary Fig. 2 with the far smaller c_2 step $\Delta c_2 = 2 \cdot 10^{-7}$. In the very left part of this figure, at $c_2 = -0.71172$, the ensemble was initialized in the $128-64-33-16-15$ state found there in the prior c_2-incremented simulation depicted in Supplementary Fig. 2. Further details are given in the figure caption.

[a)] Electronic mail: krischer@tum.de

SUPPLEMENTARY NOTE 2: REVERSE $N = 16$ SIMULATION

Fig. 3 a-b of the main article shows the transition from $8-4-4$ period-2 motion in the rotating frame via $8-4-2-2$ period-4 motion, $8-4-(4\times1)$ period-8 motion and $8-4-(4\times1)$ quasiperiodic motion to $8-4-(4\times1)$ itinerant motion. On this route, the ensemble passes through two period-doubling, a torus and a symmetry-increasing bifurcation. Supplementary Fig. 4 shows that the sequence and occurrence of bifurcations is the same if we initialize the ensemble in the $8-4-(4\times1)$ itinerant solution and move the opposite way through parameter space. There is no hysteresis here.

Only if a parameter-incremented simulation is carried out so far that the ensemble eventually jumps to a different branch will we observe hysteresis. For example, if the $N = 32$ ensemble were initialized in one of the states for $c_2 > -0.628$ in Supplementary Fig. 5 and c_2 were gradually decreased from there, the ensemble would not move back along the $16-9-7$-derived branch. Rather, it would either stay on the $16-8-8$-derived branch (if initialized at $-0.628 < c_2 - 0.627$) or on the 27–2–1–1–1-branch depicted in Supplementary Fig. 6 (if initialized in the incoherent region $c_2 > -0.627$).

SUPPLEMENTARY NOTE 3: SYMMETRY-BREAKING BIFURCATIONS TO FULL INCOHERENCE

For $\nu = 0.1$ and $\eta = 0.63$, c_2-incremented simulations like those in Fig. 3 a and Supplementary Fig. 1 do not create stable states of fully incoherent single oscillators at the end of the depicted bifurcation cascades. Instead, the ensemble will at some point jump to some other cluster-solution that is co-stable with the last step of the simulated cascade. If for example the $N = 16$ c_2-incremented simulation in Fig. 3 a is continued further, the ensemble at some point is thrown onto a $5-3-5-3$ four-cluster solution. This 5–3–5–3 is actually even co-stable with all steps of the cascade from the 8–4–4 state onward, as can be deduced from the dashed line in Fig. 2 c, where the 5–3–5–3 solution is stable below the dashed green pitchfork bifurcation line of the $8-5-3$ state. Analogously, the $N = 32$ c_2-incremented simulation in Supplementary Fig. 1, jumps to a $10-6-10-6$ solution shortly to the right of the depicted c_2 interval.

For $\eta = 0.67$, the situation is different, as no $N/2$–$N/2$-derived four-cluster solutions like the $10-6-10-6$ solu-

tion are stable beyond the end of an equivalent cluster-splitting cascade. This is illustrated in Supplementary Fig. 5, where the ensemble, when c_2-incremented from the 16–16 state at $c_2 = -0.6425$, first assumes a 16–9–7 solution and then goes through a sequence of bifurcations similar to that in Supplementary Fig. 1. As indicated by the cluster sizes in the lower part of the figure, it at some point ends up in a 16–9–(7×1) configuration. From there on, it jumps to a 16–8–4–4 solution. This solution is part of a the different somewhat longer-lasting cascade via the $16-8-8$ solution that is co-stable with the cascade via the $16-9-7$ solution that the ensemble originally went through here. Even further upward in c_2, no $N/2-N/2$-derived solutions are stable anymore, and the dynamics consequently become fully incoherent. (Whereas it might appear from the lower part of the figure as if there still exist various non-trivial clusters $N_i > 1$ for $c_2 \approx -0.626$, in particular of sizes $N_1 = 2, 3$, these are actually only precision-dependent approaches to ruins of former clusters, similar to those we observe in Fig. 4 of the main article.)

Before looking closer at those incoherent dynamics, we trace an entirely different path that also lead to them. This path starts at a $27-2-1-1-1$ solution for which the maxima of $\mathrm{Re}(w_k)$ are shown in the leftmost part of Supplementary Fig. 6 a. The trajectories of the clusters and single oscillators of this solution in the rotating frame of $\langle W \rangle$ are shown in Supplementary Fig. 7 a-b. At $c_2 \approx -0.6402$, an equivariant torus bifurcation introduces a tertiary frequency to the dynamics and explodes the cluster of 27 into a ring of single oscillators. This is reflected in the broadening of the maxima in Supplementary Fig. 6 a into continuous bands. The new solution is depicted in Supplementary Fig. 7 c-d.

After a sufficient further increase in c_2 the different variants of the emergent $2-(30\times1)$ solution collide in a symmetry-increasing bifurcation, leaving only single oscillators. (While again, Supplementary Fig. 6 b appears to detect several clusters $N_i > 1$, these, like in Supplementary Fig. 5 b, are also just cluster ruins.) Initially, however, the resultant fully incoherent attractor is still co-stable with the cascades the ensemble goes through in Supplementary Fig. 5, as evident from the fact that the extent of the c_2 axis in the two figures is the same. This only changes shortly before we reach $c_2 = -0.626$. At this value, the incoherent dynamics incorporate attractor ruins of both the formerly stable $2-(30\times1)$ in Supplementary Fig. 7 c-d and the $16-16$-derived solutions in Supplementary Fig. 5. In Supplementary Fig. 7 e, the former is mirrored by the large almost circular excursions of single oscillators away from the origin. The latter is mirrored by the two more oval blue-green loops similar to those in the solutions in Fig. 2 of the main article.

SUPPLEMENTARY NOTE 4: PATH FROM $150-50$ SOLUTION TO CHIMERA STATE

In Fig. 5 of the main article, the bifurcations from a $15-5$ two-cluster solution to a $15-(5\times1)$ chimera state are traced exactly. For $N = 200$, initializing a $150-50$ two-cluster solution at the same parameter values as in the $N = 20$ case and gradually increasing c_2 provokes a cluster-breaking cascade as well. This can be seen in Supplementary Fig. 8, which depicts two such c_2-incremented simulations with different Δc_2.

In the more coarsely incremented simulation in the upper half of the figure, the cluster of 50 clearly splits into a cluster of 26 and a cluster of 24. The more finely incremented simulation in the lower half is initialized in the resultant 150–26–24 configuration. A subsequent break-up of the cluster of 24 into a cluster of 13 and a cluster of 11 occurs in both simulations. In the lower half of the figure, we can also distinguish a break-up of the cluster of 11 into a cluster of 6 and a cluster of 5, as well as the later collapse of the cluster of five.

Further increases in c_2 causes the ensemble to jump between various parallel multi-cluster solution branches that all have in common that the large cluster of 150 oscillators remains intact. Eventually, the last of these multi-cluster solutions undergoes a symmetry-increasing bifurcation, creating a $150-(50\times1)$ chimera state.

For even greater values of c_2, the large cluster absorbs some of the single oscillators, increasing the imbalance between synchronized and single oscillators. Ultimately, the large cluster also collapses, leading to a state of full incoherence.

SUPPLEMENTARY NOTE 5: α-INCREMENTED SIMULATIONS BEHIND THE SCHEMATICS IN FIG. 7

In order to chart the cluster-splitting cascades and general sequences of bifurcations in the ensemble of globally coupled pitchfork maps, we performed various α-incremented simulations for fixed values of β and different ensemble sizes N. The outcome of one such α-incremented simulation for $N = 16$ is shown in Supplementary Fig. 9, where the upper part of the figure shows the range of values reached by any of the maps for each value of α. This diagram clearly shows two subsequent period-doublings of the maps inhabiting positive values $y_k(n) > 0$, followed by a Neimark-Sacker, a symmetry-increasing bifurcation and a final subcritical transition to a different solution. The lower part of the figure shows that the two period-doublings are accompanied by cluster splittings, first from an $8-8$ to an $8-4-4$ and then to an $8-(4\times2)$ configuration. The symmetry-increasing bifurcation is accompanied by the appearance of single, unclustered maps.

Supplementary Fig. 10 a-b shows an analogous depiction of a similar α-incremented simulation for $N = 128$,

initialized in the $64-33-31$ configuration. The simulation begins with three clearly discernable cluster-splitting period-doubling bifurcations, reaching a 64–33–16–8–4–3 configuration somewhat above $\alpha = 0.87$. These bifurcations are followed by a string of less easily identifiable transitions between different solutions. Above $\alpha \approx 0.877$, persistent clusters clearly coexist with loose and temporary agglomerations, a sign of itinerant dynamics for large N, as also seen in the Stuart-Landau ensemble. Around $\alpha \approx 0.883$, the cluster of 64 is for a certain α interval the only persistent cluster, signifying a 64–(64×1) chimera state. In Supplementary Fig. 10 c, the cluster solution found at $\alpha \approx 0.87$ in Supplementary Fig. 10 a-b is incremented in smaller steps of α. This shows that the initial cluster-halving cascade indeed also reaches a $64-33-16-8-4-2-1$ configuration before the larger clusters are broken.

SUPPLEMENTARY NOTE 6: PREMATURELY ENDING PITCHFORK-MAP CLUSTER-SPLITTING CASCADE FOR N=256

In the case of $N = 256$, the period-doubling cluster-splitting bifurcation of the $N/2$–$N/2 = 128$–128 solution also produces a $128-65-63$ solution. If this solution is traced upward in α, the cluster of 63 is split into smaller clusters of 32 and 31, analogous to the development of most of the cluster-splitting cascades described above. However, the next bifurcation encountered is not another period-doubling, but an equivariant pitchfork splitting the cluster of 65. Supplementary Fig. 11 a clearly shows that thereby, the hitherto period-2 trajectories of the maps in the cluster of 65 (depicted in green) are replaced by period-4 trajectories. The result, a 128–34–31–32–31 period-4 solution, reminds us of the $8-(4\times 2)$ period-4 solution produced in the second period-doubling of the $N = 16$ ensemble in Supplementary Fig. 9. Like in that simulation, the next bifurcation in the $N = 256$ ensemble is a Neimark-Sacker bifurcation, followed by a symmetry-increasing bifurcation, as evident from the coexistence of persistent and temporary clusters from somewhat above $\alpha = 0.88$. Another symmetry-increasing bifurcation creates a $128-(128\times 1)$ balanced chimera state.

SUPPLEMENTARY NOTE 7: SPATIALLY AVERAGED ELLIPSOMETRIC SIGNALS

Supplementary Fig.12 shows the timeseries of the spatial average of the ellipsometric signal normalised to its maximum ξ_{av}/ξ_{av}^{max}. The order of the states is the same as in Fig. 9 of the main manuscript, i.e., Supplementary Fig. 12 a depicts the spatially averaged ellipsometric signel for the antiphase state, b for the subharmonic clustering, c for the chimera and d for the turbulent state. In each case, the periodic oscillation of the average signal is conserved, similar to the conservation law for the average value of the globally coupled Stuart-Landau oscillators. This justifies subtracting the homogeneous mode to enter a rotating frame of reference.

SUPPLEMENTARY NOTE 8: c_2-INCREMENTED SIMULATION FOR ODD $N = 63$

Whereas this article focuses on even ensemble sizes, its findings seem to be valid for odd N as well. In particular, Supplementary Fig. 13 shows that both the cluster-halving cascade and a subsequent symmetry-increasing bifurcation can be observed for $N = 63$.

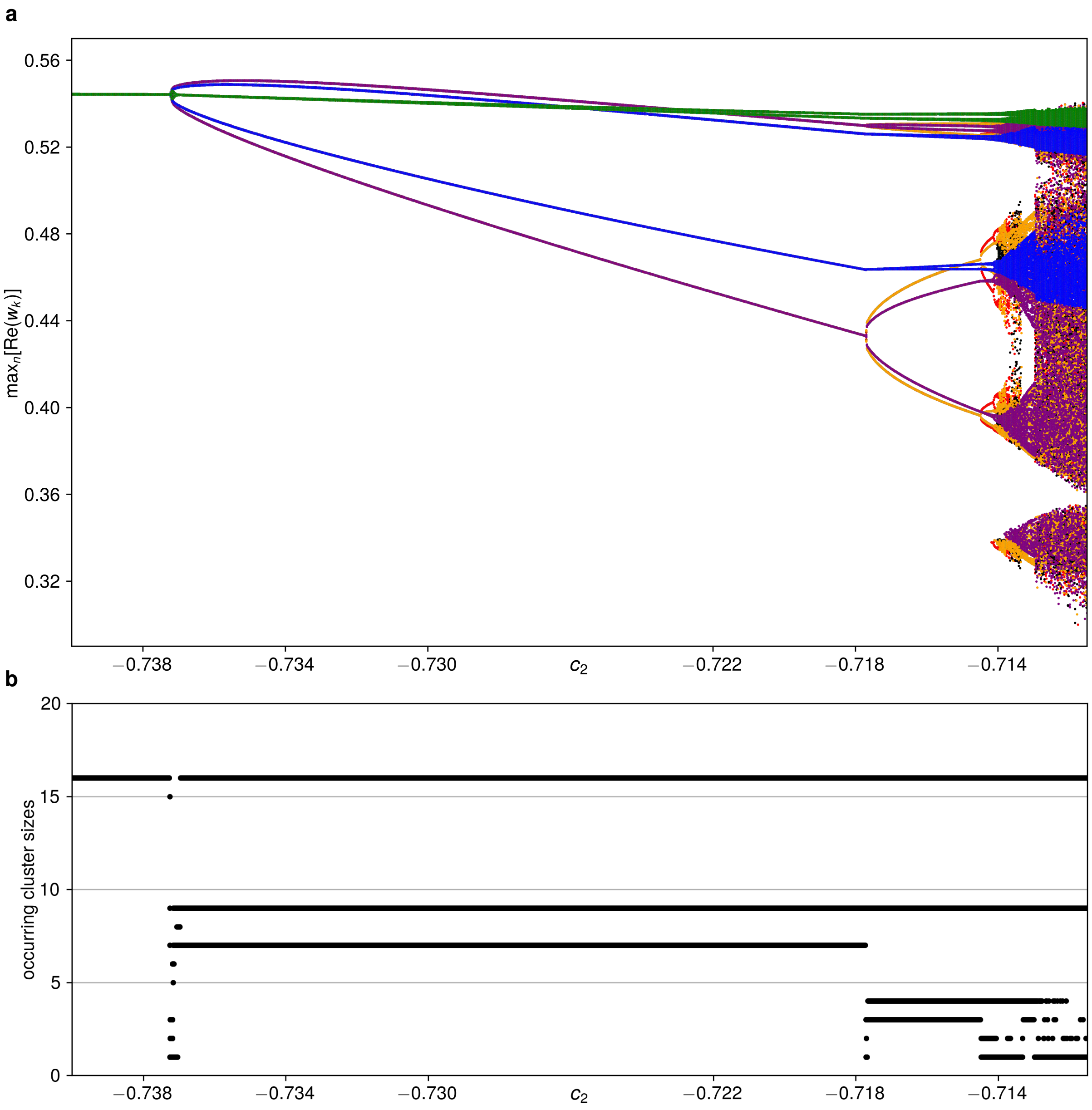

Supplementary Fig. 1. **Cluster-splitting cascade and ensuing bifurcations for** $N = 32$. **a**, All occurring maxima of the rotating-frame real parts $\mathrm{Re}(w_k)$ of all clusters and single oscillators against c_2 as c_2 is gradually increased at a rate of $\Delta c_2 = 2 \cdot 10^{-5}$ every 10^4 time steps for $N = 32$, $\nu = 0.1$ and $\eta = 0.63$. Oscillators are colored by the clusters to which they belong in the cluster-splitting cascade. Initially, there are two clusters of 16, reaching a single maximum shown in green. At $c_2 \approx -0.737$, one of these clusters splits up into a cluster of nine, shown in blue, and a cluster of seven, shown in purple, that both are period-2. At $c_2 \approx -0.7177$, the cluster of seven splits up into a cluster of four (purple) and a cluster of three (yellow). At $c_2 \approx -0.7145$, the cluster of three splits up into a cluster of two (yellow) and a single oscillator (red). When the cluster of two is destroyed, the two resulting single oscillators are shown in red and black. At higher c_2 values, additional single oscillators retain the color of the cluster to which they belonged in the $16-9-4-1-1-1$ solution. **b**, Cluster sizes at each value of c_2 during the c_2-incremented simulation in **a**. Calculations are based on the cross correlations of trajectories (in the non-rotating frame) over the last 800 time steps of simulation at each c_2 value and a threshold of $\varepsilon = 10^{-8}$. See Methods section.

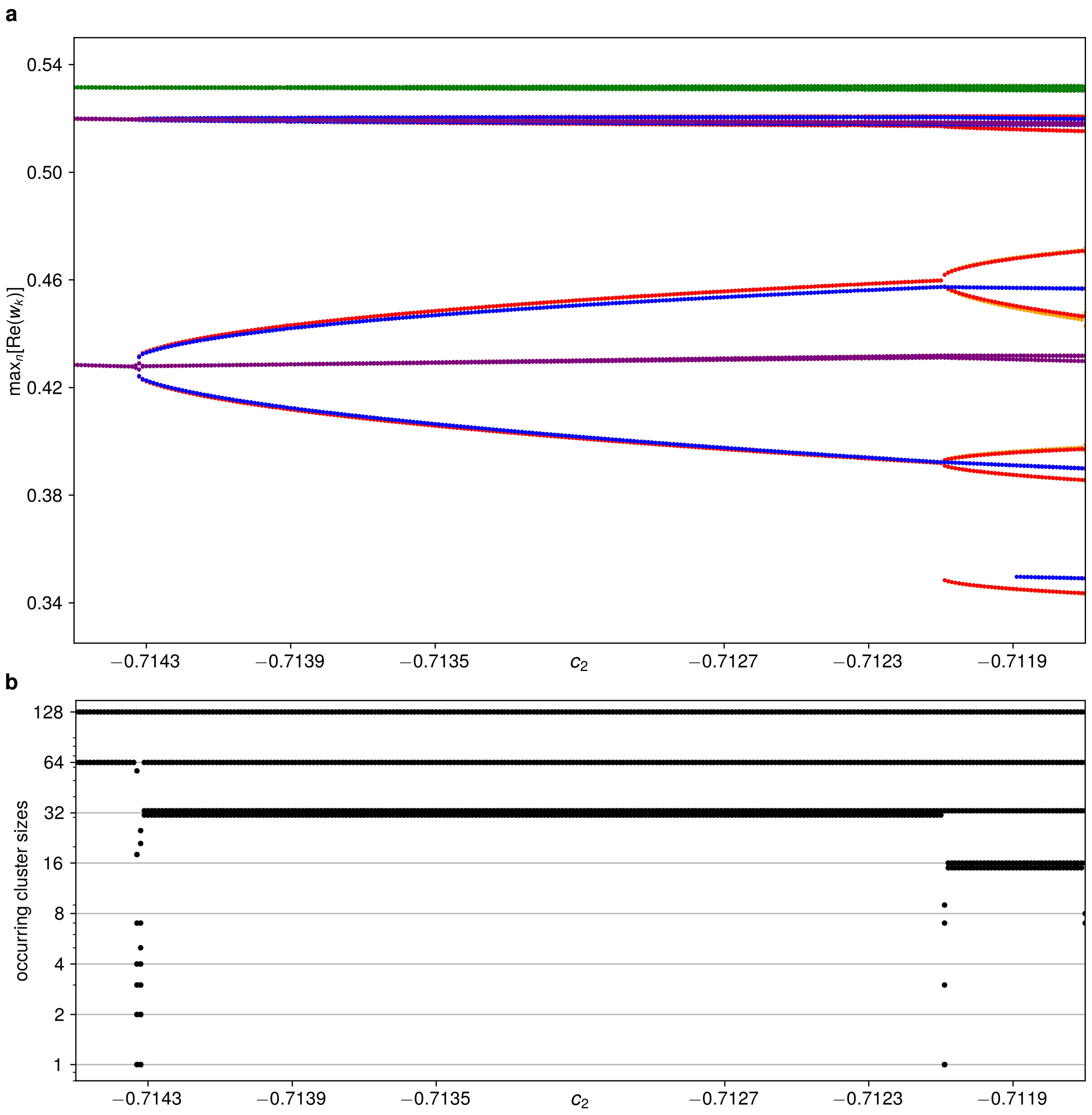

Supplementary Fig. 2. **First cluster-splitting bifurcations for** $N = 256$**. a**, All occurring maxima of the real parts of all clusters and single oscillators against c_2 as c_2 is gradually increased at a rate of $\Delta c_2 = 10^{-5}$ every $2 \cdot 10^4$ time steps for $N = 256$, $\nu = 0.1$ and $\eta = 0.63$. The simulation is initialized in the $128-64-64$ solution at $c_2 = -0.7145$. Oscillators are colored by the clusters to which they belong in the final $128-64-33-16-15$ solution: In the leftmost part of the figure, the maxima of the cluster of 128 are shown in green, the maxima of the two clusters of 64 in purple. At $c_2 \approx -0.7143$, one of these clusters splits up into a cluster of 33 (blue) and a cluster of 31 (red). At $c_2 \approx -0.7121$, the cluster of 31 splits up into a cluster of 16 (red) and a cluster of 15 (yellow). **b**, Cluster sizes at each value of c_2 during the c_2-incremented simulation in **a**. Calculations are based on the cross correlations of trajectories (in the non-rotating frame) over the last 800 time steps of simulation at each c_2 value and a threshold of $\varepsilon = 10^{-8}$. See Methods section. The vertical axis scales logarithmically.

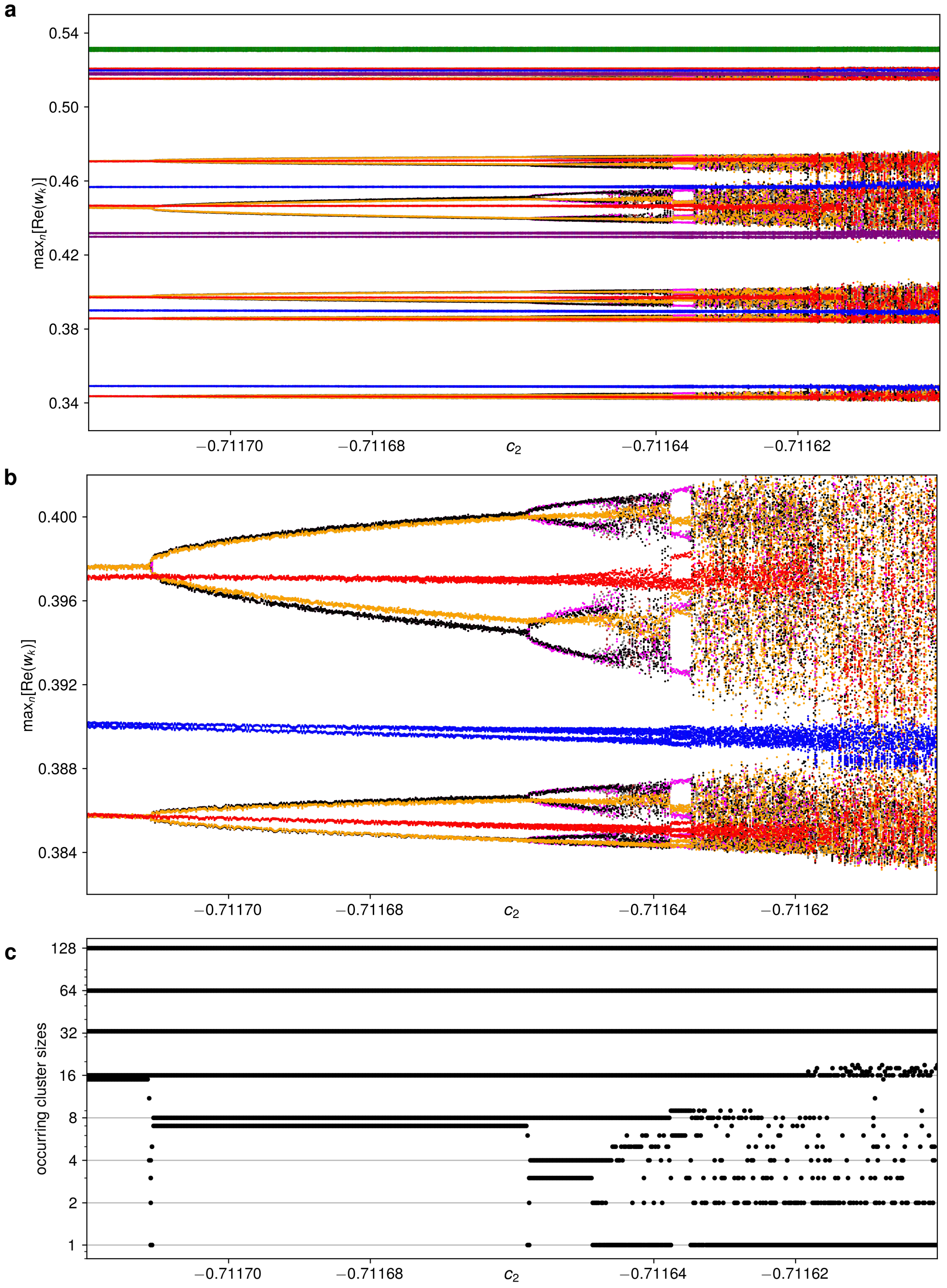

Supplementary Fig. 3. **Continued cluster-splitting cascade and ensuing bifurcations for** $N = 256$**. a**, All occurring maxima of the real parts of all clusters and single oscillators against c_2 as c_2 is gradually increased at a rate of $\Delta c_2 = 2 \cdot 10^{-7}$ every $2 \cdot 10^4$ time steps for $N = 256$, $\nu = 0.1$ and $\eta = 0.63$. The simulation is initialized in the 128−64−33−16−15 solution found at $c_2 = -0.71172$ in Supplementary Fig. 2. In the leftmost part of the figure, the maxima of the clusters of 128, 64, 33, 16 and 15 are shown in green, purple, blue, red and yellow, respectively. At $c_2 \approx -0.71171$, the cluster of 15 splits up into a cluster of eight (yellow) and a cluster of seven (black). At $c_2 \approx -0.71166$, the cluster of seven splits up into clusters of four and three (in black and pink, respectively). At $c_2 \approx -0.71165$, the cluster of three splits up into a cluster of two (pink) and a single oscillator (grey), manifest only in the appearance of additional grey dots and easier seen in **c**. At higher c_2 values, additional single oscillators retain the color of the cluster to which they belonged in the 128−64−33−16−8−4−2−1 solution. **b**, Magnified view of the maxima $0.382 < \max_n[\mathrm{Re}(w_k)] < 0.404$ in **a**. **c**, Cluster sizes at each value of c_2 during the simulation in **a,b**. Calculations are based on the last 800 time steps of simulation at each c_2 value and a threshold of $\varepsilon = 10^{-8}$. See Methods section. The vertical axis scales logarithmically.

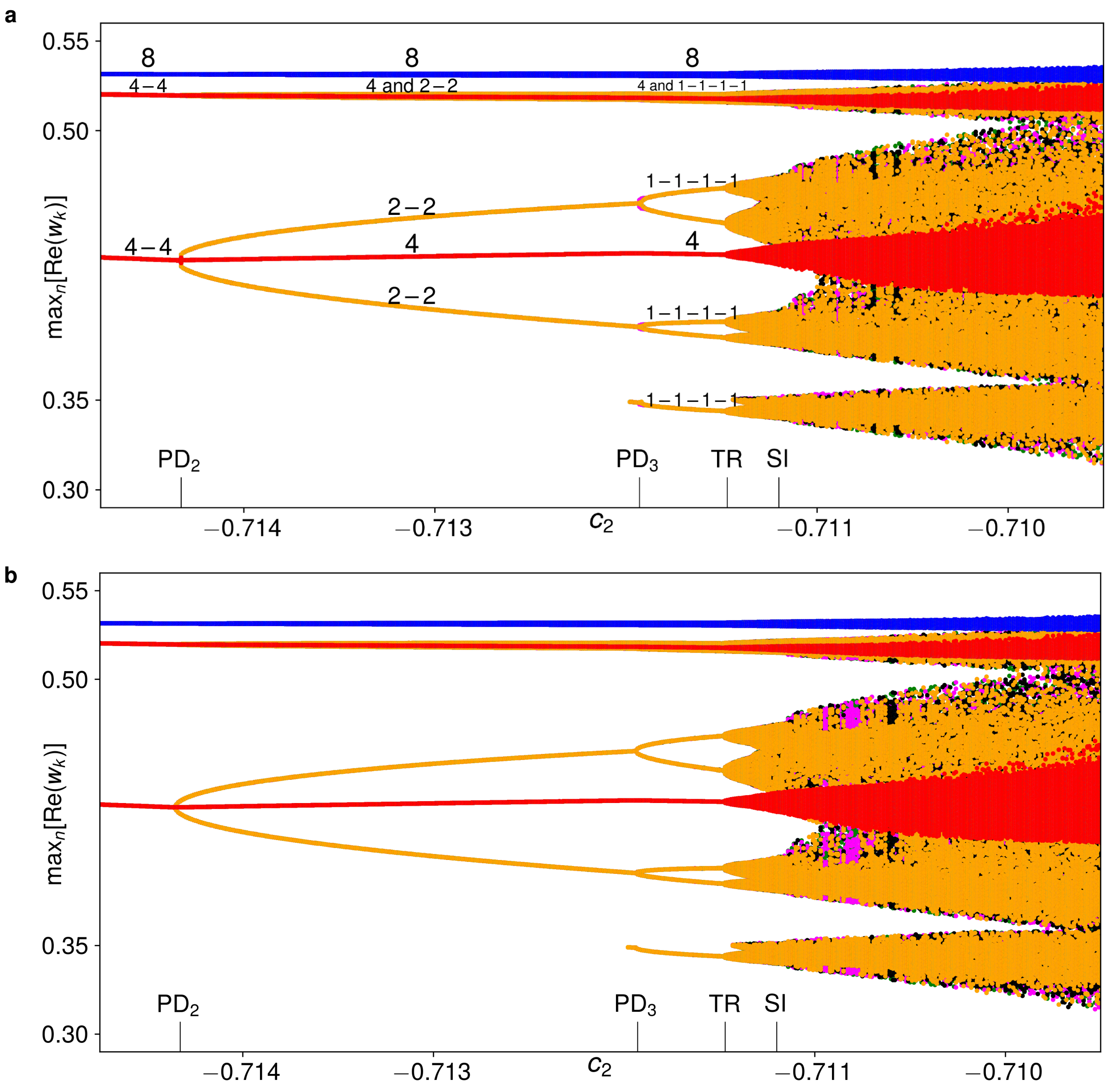

Supplementary Fig. 4. **c_2-incremented and decremented simulations for $N = 16$. a**, Maxima of $\mathrm{Re}(w_k)$ against c_2 as c_2 is gradually increased at a rate of $\Delta c_2 = 10^{-5}$ every $4 \cdot 10^4$ time steps for $\eta = 0.63$. This figure is also included in the main manuscript as Fig. 3 a. Labels on the figure mark the clusters reaching the different maxima as the solution changes from 8—4—4 via 8—4—2—2 to 8—4—(4×1). Labels on the abscissa mark occurring period-doubling (PD), torus (TR) and symmetry-increasing (SI) bifurcations. The additional smallest yellow maximum appearing at $c_2 \approx -0.712$ is caused by the continuous deformation of the oscillator trajectories and not by a bifurcation. At $c_2 \approx -0.7115$, the torus bifurcation to three-frequency dynamics manifests itself in a distinct broadening of the formerly discrete maxima. **b**, Maxima of $\mathrm{Re}(w_k)$ as c_2 is gradually *decreased* at a rate of $\Delta c_2 = -10^{-5}$ every $4 \cdot 10^4$ time steps after initializing the ensemble at the right edge of **a**. In the itinerant parameter region $c_2 > -0.7112$, recorded maxima and transient clusters differ for the two solutions, but the qualitative dynamics are the same. Moreover, all indicated bifurcations (PD, TR and SI) occur at the respective same parameter values.

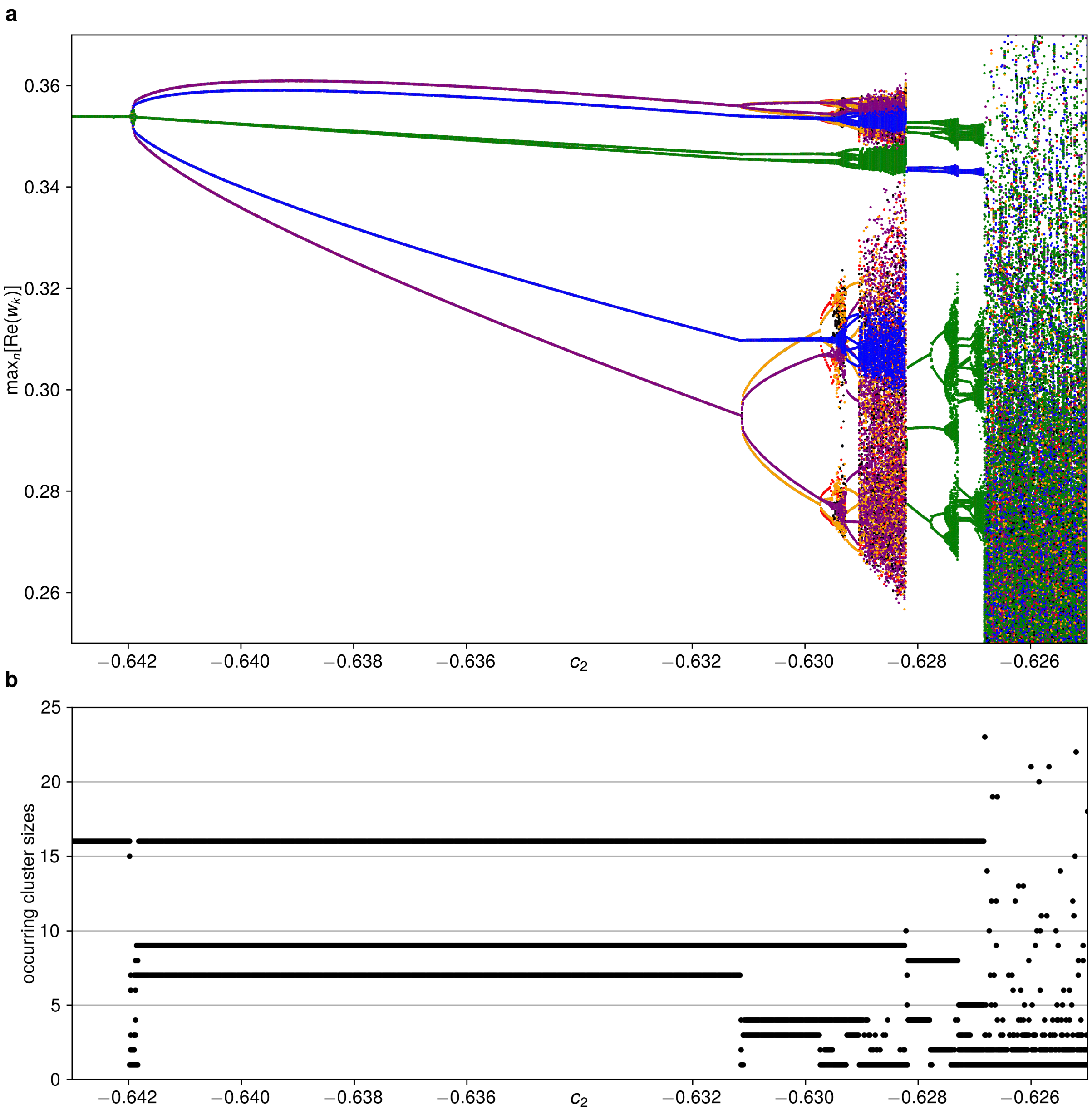

Supplementary Fig. 5. **Cluster-splitting cascade and ensuing bifurcations for** $N = 32$ **and** $\eta = 0.67$**, starting at** 16−16. **a**, All occurring maxima of the real parts $\mathrm{Re}(w_k)$ of all clusters and single oscillators against c_2 as c_2 is gradually increased at a rate of $\Delta c_2 = 2 \cdot 10^{-5}$ every 10^4 time steps. Oscillators are colored by the clusters to which they belong in the cluster-splitting cascade taking up most of the depicted range of c_2: Initially, there are two clusters of 16, reaching a single maximum shown in green. At $c_2 \approx -0.642$, one of these clusters splits up into a cluster of nine (blue) and a cluster of seven (purple), that both are period-2. At $c_2 \approx -0.6315$, the cluster of seven splits up into a cluster of four (purple) and a cluster of three (yellow). At $c_2 \approx -0.7298$, the cluster of three splits up into a cluster of two (yellow) and a single oscillator (red). When the cluster of two is destroyed, the two resulting single oscillators are shown in red and black. At higher c_2 values, additional single oscillators retain the color of the cluster to which they belonged in the 16−9−4−1−1−1 solution. At $c_2 \approx -0.6282$, the ensemble jumps to the 16−8−4−4 solution. At $c_2 \approx -0.2668$, the cluster of 16 is destroyed and there are henceforth only single oscillators. In this irregular regime, the maxima of $\mathrm{Re}(w_k)$ reach as far down as $\max[\mathrm{Re}(w_k)] = -0.8$. These maxima have been cut off for a clearer view of the dynamics at lower c_2 values. This same final state is also reached from the very different starting point in Supplementary Figure 6. **b**, Cluster sizes at each value of c_2 during the c_2-incremented simulation in **a**. Calculations are based on cross correlations of trajectories (in the non-rotating frame) over the last 800 time steps of simulation at each c_2 value and a threshold of $\varepsilon = 10^{-8}$. See Methods section.

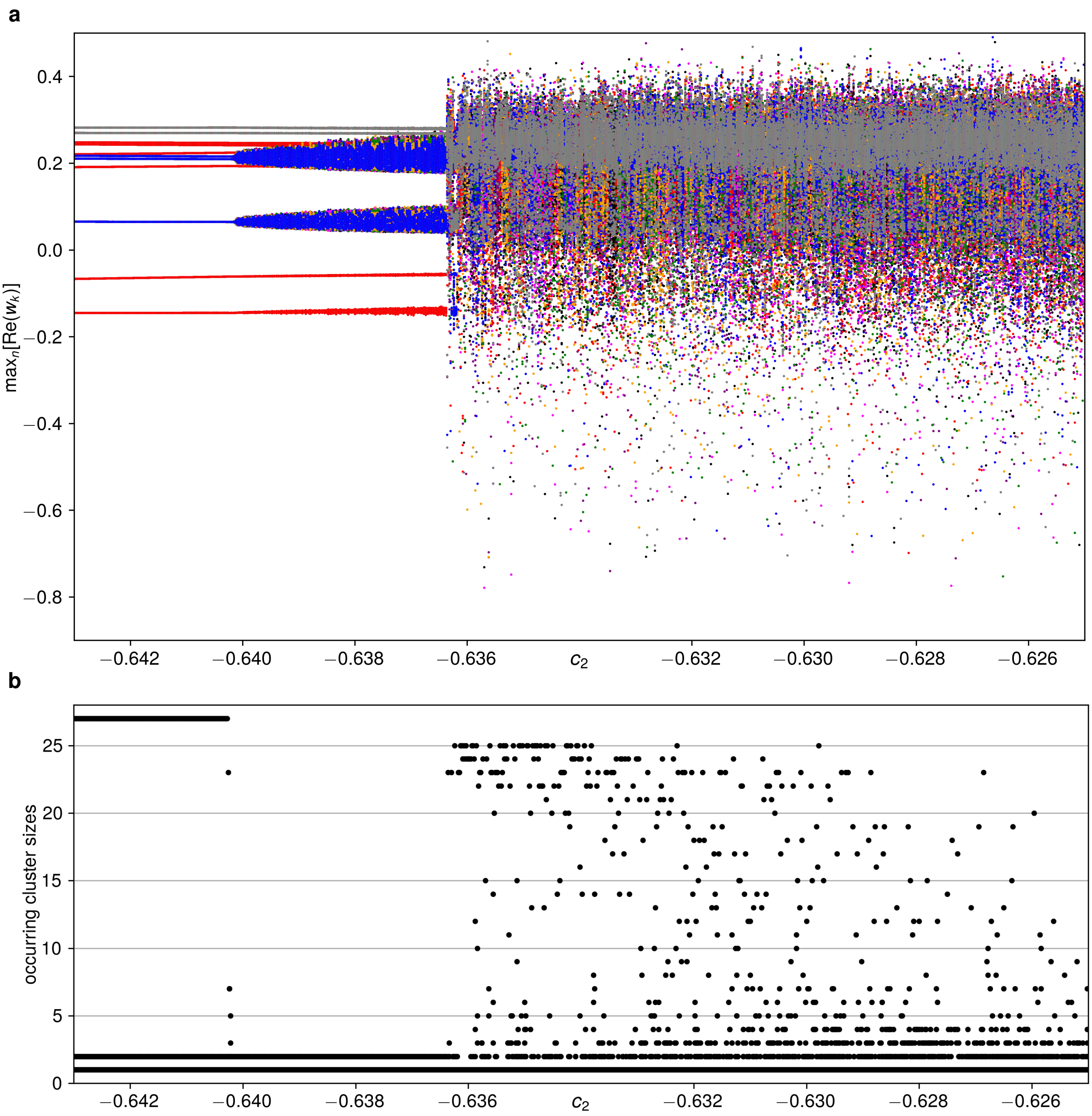

Supplementary Fig. 6. **Bifurcations encountered for** $N = 32$ **and** $\eta = 0.67$**, when starting at** $27-2-1-1-1$ **and increasing** c_2**. a**, All occurring maxima of the real parts $\mathrm{Re}(w_k)$ of all clusters and single oscillators against c_2 as c_2 is gradually increased at a rate of $\Delta c_2 = 2 \cdot 10^{-5}$ every 10^4 time steps for $N = 32$, $\nu = 0.1$ and $\eta = 0.67$. The initial state is a $27-2-1-1-1$ quasiperiodic solution, with maxima of the cluster of 27 shown in blue, those of the cluster of two in grey and those of the three single oscillators, which all pursue the same solution, in red. At $c_2 \approx -0.6402$, a torus bifurcation breaks the cluster of 27 into single oscillators, and broadens the formerly discrete maxima into continuous ranges. At $c_2 \approx -0.6364$, this solution undergoes a symmetry-increasing collision to only single oscillators. This final state is also reached from the very different starting point in Supplementary Figure 6. **b**, Cluster sizes at each value of c_2 during the c_2-incremented simulation in **a**. The further c_2 is increased away from the symmetry-increasing bifurcation, the less distinct do the ruins of the former ordered state become and the less likely are many of the oscillators to cluster at any given time. Calculations are based on cross correlations of trajectories (in the non-rotating frame) over the last 800 time steps of simulation at each c_2 value and a threshold of $\varepsilon = 10^{-8}$. See Methods section.

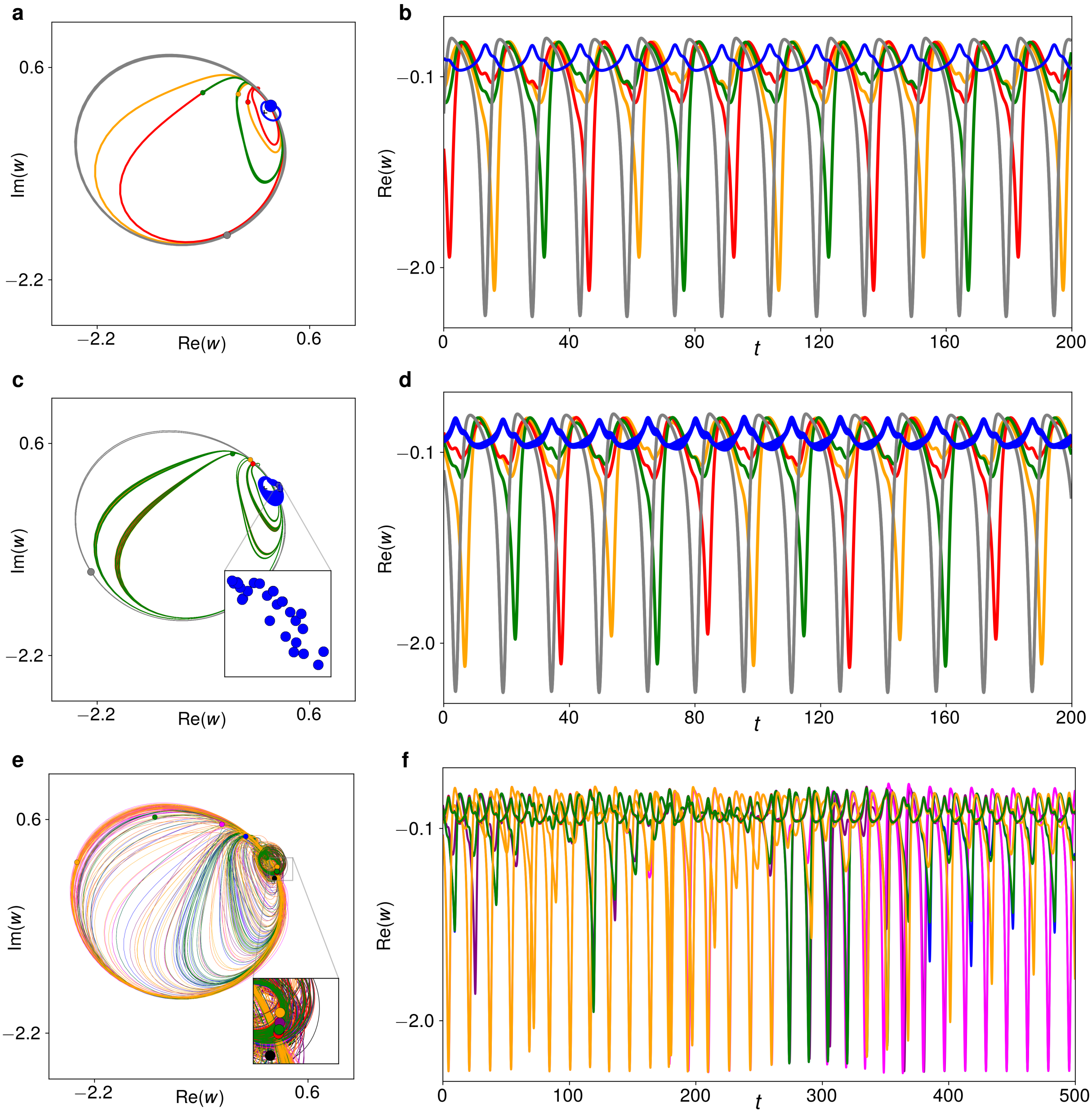

Supplementary Fig. 7. **Solutions encountered along the simulation in Supplementary Fig. 6.** **a**, Complex-plane portrait of the 27−2−1−1−1 solution at $c_2 = -0.642$. The cluster of 27 two is shown in blue, the cluster of two in grey and the three single oscillators in red, green and yellow, respectively. In order not to obscure its trajectory, the blue dot marking the instantaneous location of the cluster of 27 is smaller than 27 times as large as the dots marking the single oscillators. **b**, Time series of the real part of each cluster and oscillator in **a**. **c,d**, Like **a,b** for the $2-(30\times 1)$ state found at $c_2 = -0.638$, past the torus bifurcation in Supplementary Figure 6. Here, the single oscillators from the former cluster of 27 are all still plotted in blue. **e,f**, Solution without permanent clusters at $c_2 = -0.625$. Here, eight different colors are arbitrarily used to depict the $N = 32$ different oscillator trajectories, with four oscillators of each color. Thus, line segments of the same color do not necessarily belong to the same oscillator. In the depicted interval, the ensemble has moved both close to ruins of the former $27-2-1-1-1$ branch and to ruins of the $16-16$ branch, as indicated by the two blue-green loops similar to those of the solutions in Fig. 2 of the main article.

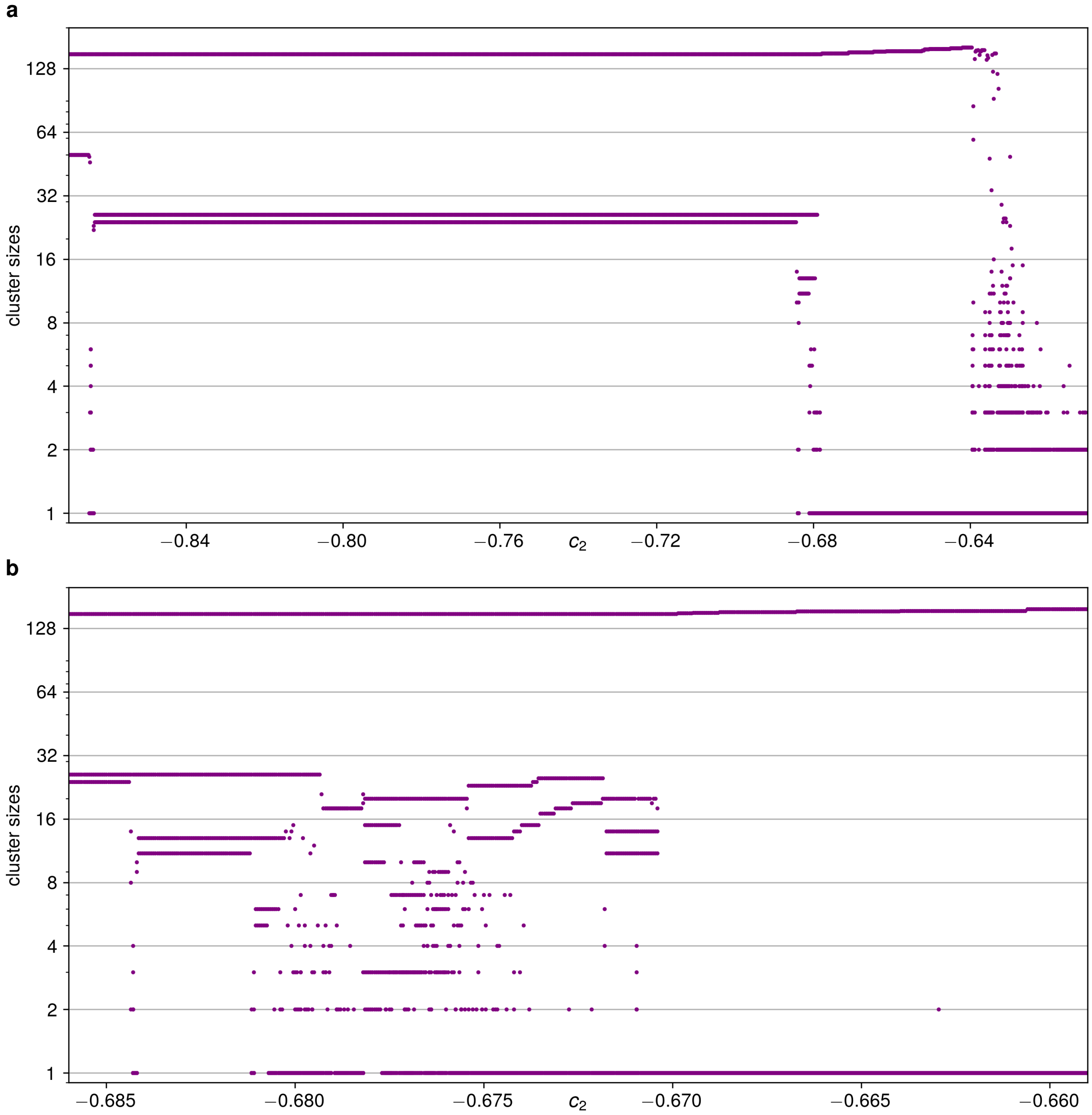

Supplementary Fig. 8. **Cluster sizes recorded along the path from a** 150–50 **solution to chimera state. a**, Occurring cluster sizes among $N = 200$ oscillators as c_2 is gradually incremented by $\Delta c_2 = 2 \cdot 10^{-4}$ every 2100 time steps at $\nu = 0.1$ and $\eta = 0.67$. Two oscillators are counted as being in the same cluster if their correlation distance in the interval from $t = 2000$ to $t = 2100$ at the relevant value of c_2 is less than 10^{-10}. Note that the vertical axis scales logarithmically. **b**, Analogue to **a**, but for a shorter c_2 interval and with a smaller increment $\Delta c_2 = 5 \cdot 10^{-5}$ and longer simulation time of 4200 time steps at each c_2 value. In both **a** and **b**, the initial cluster splittings are accompanied by transient many-cluster solutions that are not allowed to subside before the number of clusters is calculated. This causes the temporary increase in recorded small clusters at the beginning of each step of the cascade. Some of the multi-cluster solutions at higher values of c_2 could thus also be transients.

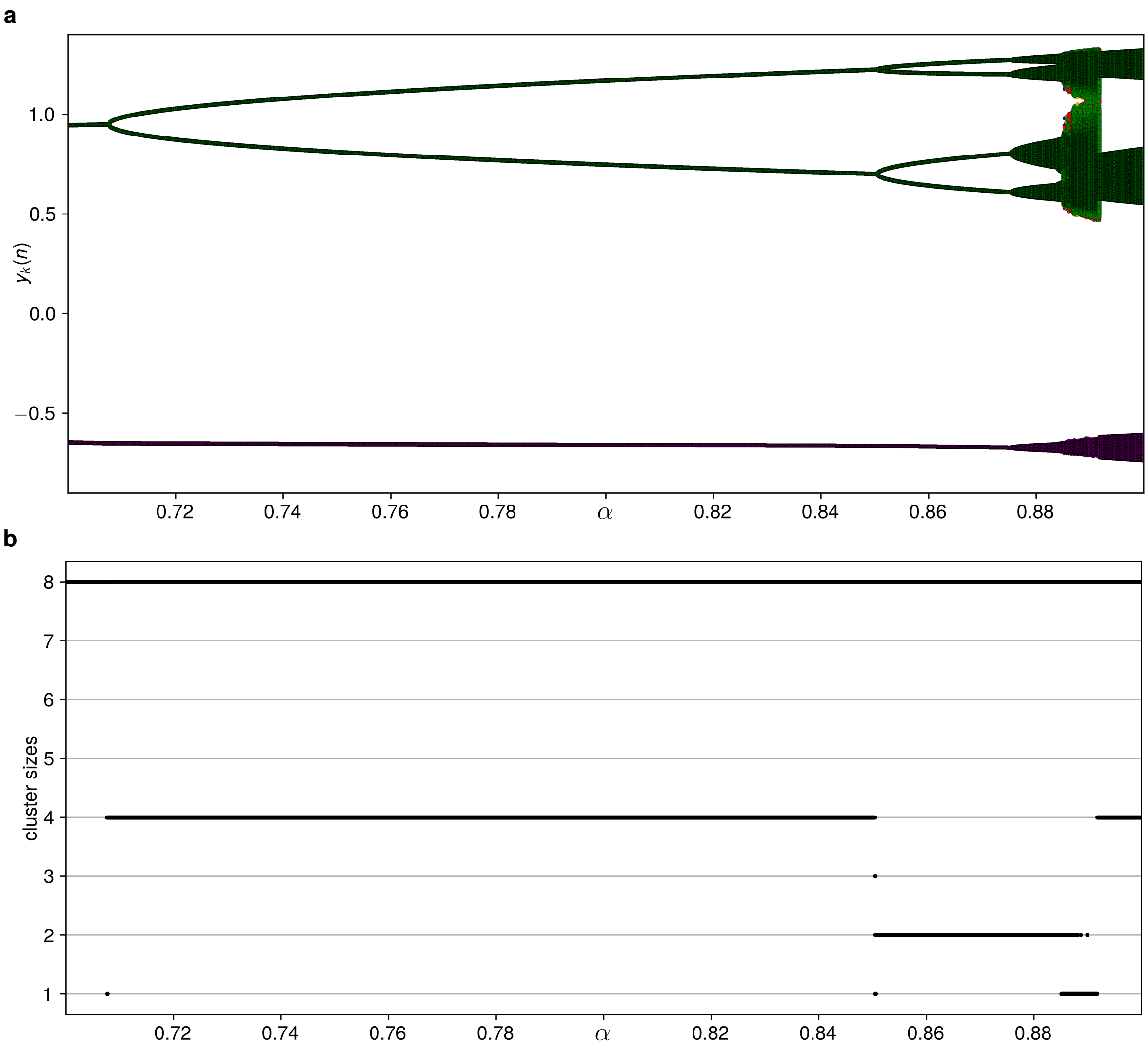

Supplementary Fig. 9. **α-incremented simulation of $N = 16$ globally coupled pitchfork maps for $\beta = 0.15$.** **a**, All occurring values $y_k(n)$ of all clusters and single maps against α as α is gradually increased at a rate of $\Delta\alpha = 10^{-4}$ every 10^4 iterations of the maps. The values $y_k(n) < -0.5$ are reached by a non-splitting cluster of eight. The leftmost values ≈ 1.0 are reached by the other initial cluster of eight. At $\alpha \approx 0.71$, this cluster splits into two smaller of size four, both pursuing the same period-2 trajectory. At $\alpha \approx 0.85$, each of these clusters splits into two even smaller clusters of size two, henceforth pursuing the same period-4 trajectory. At $\alpha \approx 0.875$, a Neimark-Sacker bifurcation takes place, not affecting the cluster sizes. Somewhat above $\alpha = 0.88$, the clusters of two are broken in a symmetry-increasing bifurcation, resulting in itinerant motion. Even further upward in α, the system transitions to a $8-4-4$ quasiperiodic solution. **b**, Cluster sizes at each value of α during the α-incremented simulation in **a**. To calculate the cluster sizes for a certain value of α, the time series containing the 2000 last increments of each map at that value of α are compared pairwise. Two maps are said to be in the same cluster if the Euclidean distance between their 2000-dimensional time vectors is less than $\varepsilon = 10^{-4}$.

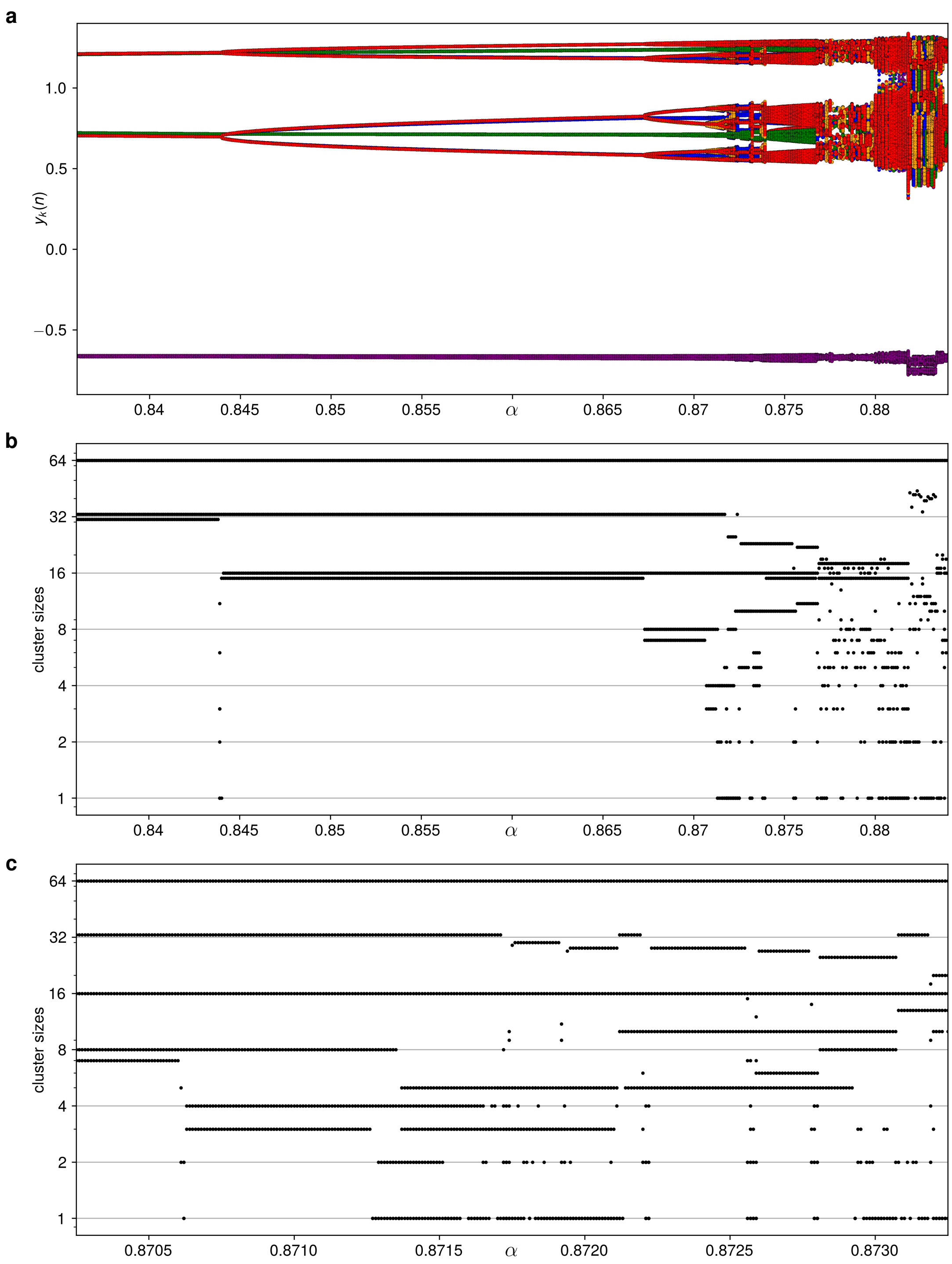

Supplementary Fig. 10. **α-incremented simulation of N = 128 globally coupled pitchfork maps for β = 0.15. a**, All occurring values $y_k(n)$ of all clusters and single maps as α is gradually increased at a rate of $\Delta\alpha = 10^{-4}$ every 10^4 iterations n of the maps. The initial cluster-size configuration is 64–33–31. The values $y_k(n) < -0.5$ are reached by the cluster of 64. In the very left, values of the cluster of 33 are shown in green, those of the cluster of 31 in red. At $\alpha \approx 0.844$, the cluster of 31 splits into a cluster of 16 (blue) and a cluster of 15 (red). At $\alpha \approx 0.867$, the cluster of 15 splits into a cluster of 8 (red) and a cluster of 7 (yellow). At $\alpha \approx 0.870$, the cluster of 7 splits into a cluster of 4 and a cluster of 3. **b**, Cluster sizes at each value of α during the simulation in **a**. Cluster sizes are calculated like in Supplementary Fig. 9. **c**, Cluster sizes during a more finely α-incremented simulation, initialized in the $64-33-16-8-7$ solution from **c**, with α increased by $\Delta\alpha = 10^{-5}$ every 10^4 iterations n of the maps.. This shows that a stable $64-33-16-8-4-2-1$ is indeed reached around $\alpha = 0.8713$ before the larger clusters are split.

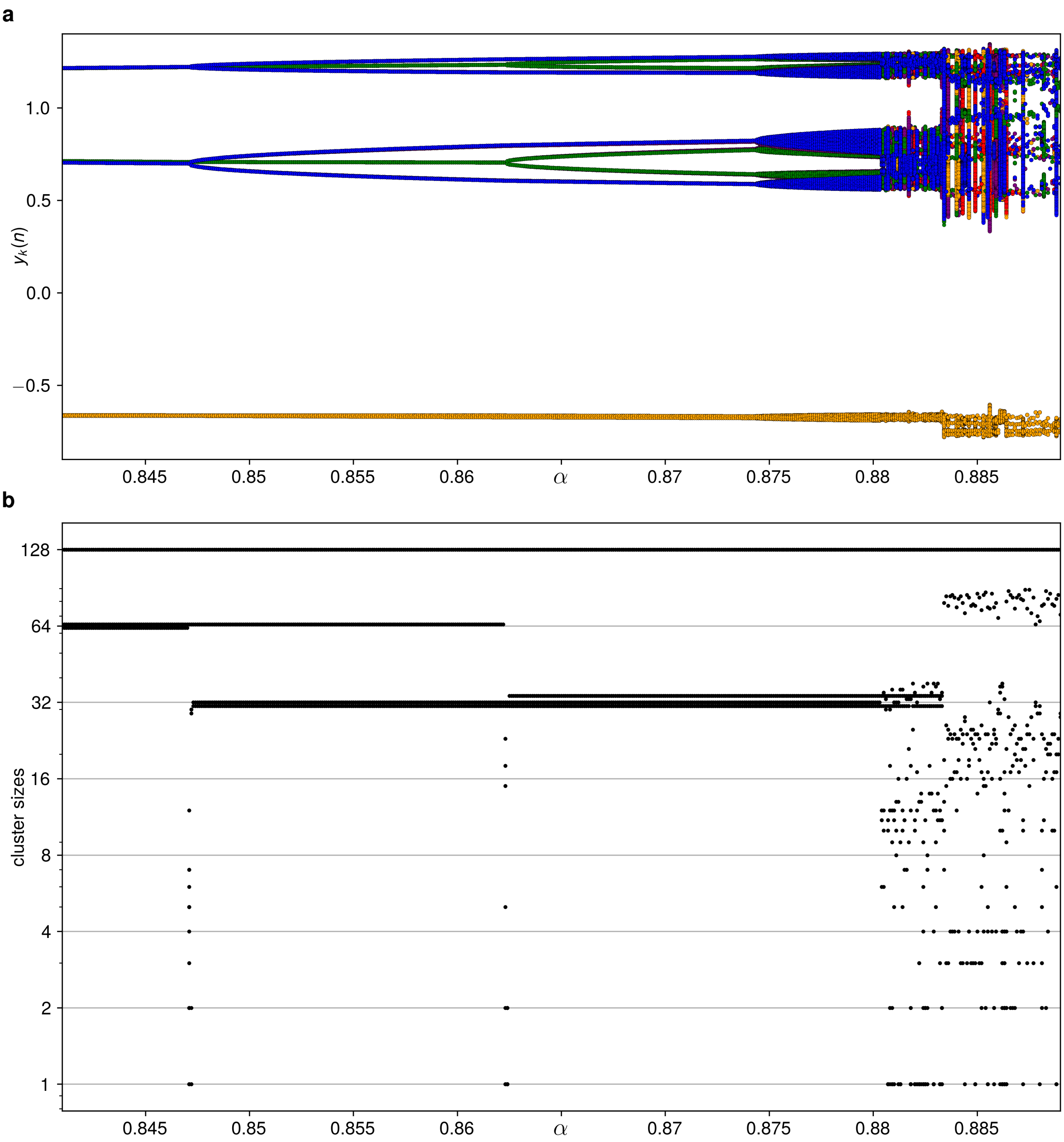

Supplementary Fig. 11. **α-incremented simulation of $N = 256$ globally coupled pitchfork maps for $\beta = 0.15$.** **a**, All occurring values $y_k(n)$ of all clusters and single maps as α is gradually increased at a rate of $\Delta\alpha = 10^{-4}$ every 10^4 iterations n of the maps. The initial cluster-size configuration is $128-65-63$. The values $y_k(n) < -0.5$ are reached by the cluster of 128. In the very left, values of the cluster of 65 are shown in green, those of the cluster of 63 in blue. At $\alpha \approx 0.847$, the cluster of 63 splits into a cluster of 32 (blue) and a cluster of 31 (purple). At $\alpha \approx 0.862$, the cluster of 65 splits into a cluster of 34 (green) and a cluster of 31 (red), without increasing the overall period. The next bifurcation is a Neimark-Sacker bifurcation at $\alpha \approx 0.874$. **b**, Cluster sizes at each value of α during the simulation in **a**. Here it becomes apparent that the Neimark-Sacker bifurcation is followed by two symmetry-increasing bifurcations at higher values of α, one wherein the cluster of 32 and a cluster of 31 is destroyed, and another wherein the other smaller clusters are broken, resulting in a $128-(128\times1)$ balanced chimera state. Cluster sizes are calculated like in Supplementary Fig. 9.

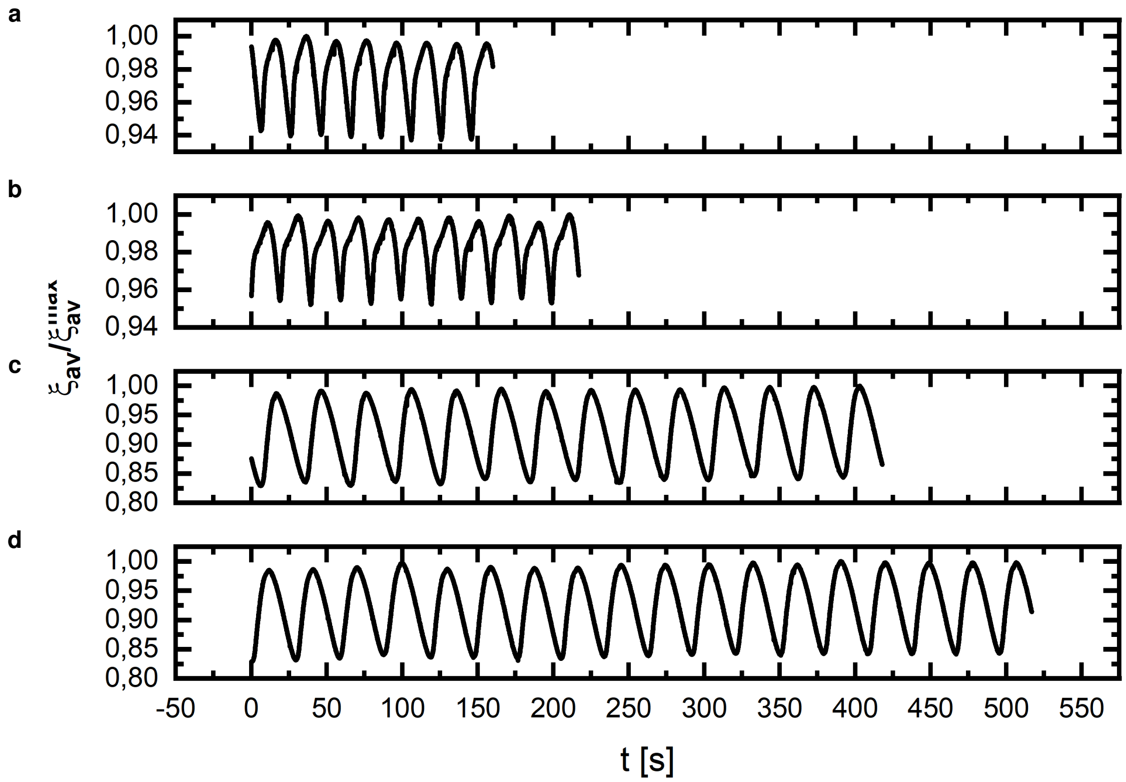

Supplementary Fig. 12. **Time-series of the spatial average of the ellipsometric signal**, normalised to its maximum $\xi_{\mathrm{av}}/\xi_{\mathrm{av}}^{\max}$ for **a** the anti-phase state, **b** the sub-harmonic cluster state, **c** the chimera state and **d** the turbulent state.

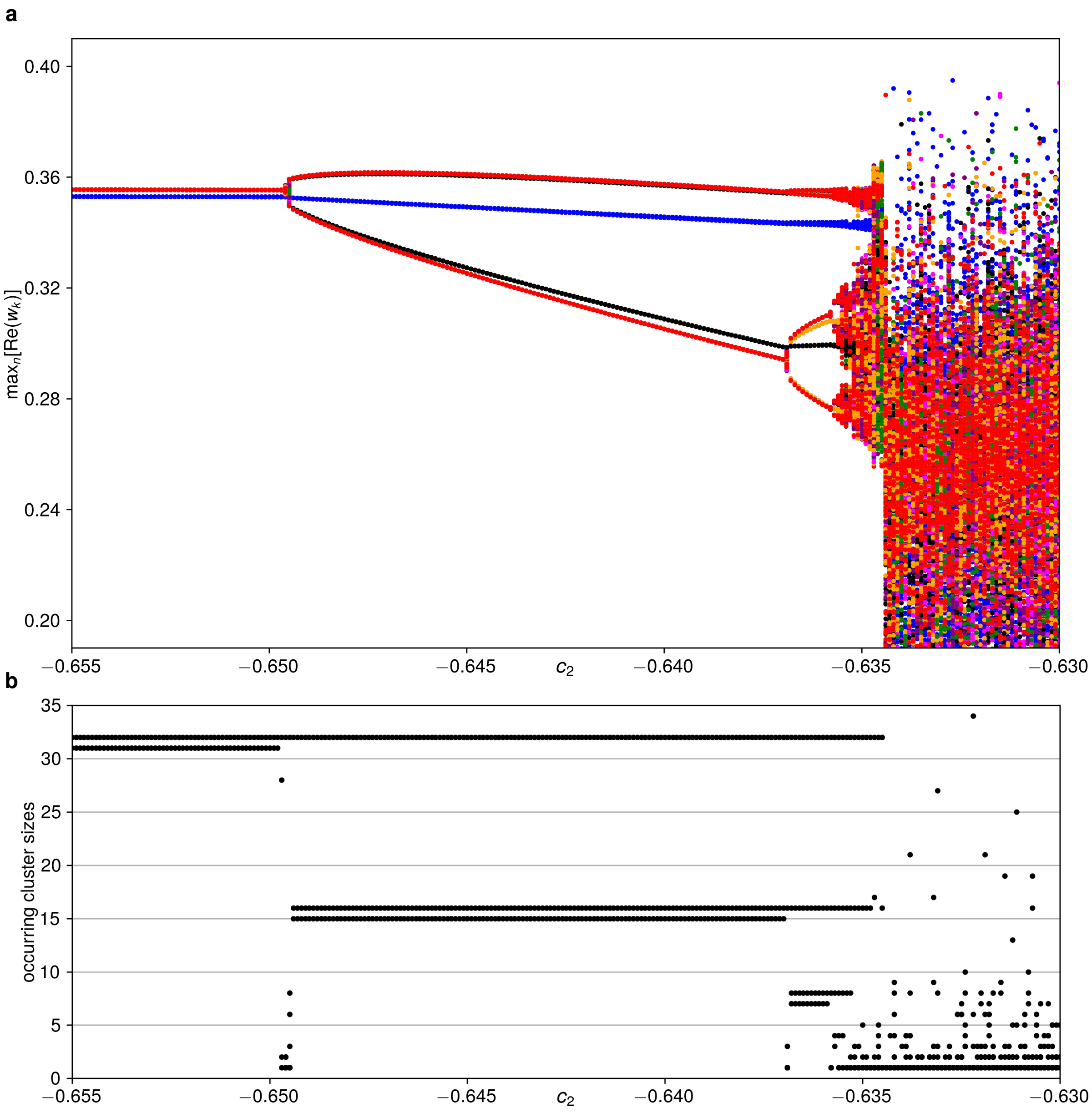

Supplementary Fig. 13. **Cluster-splitting cascade and ensuing bifurcations for** $N = 63$. **a**, All occurring maxima of the rotating-frame real parts $\mathrm{Re}(w_k)$ of all clusters and single oscillators against c_2 as c_2 is gradually increased at a rate of $\Delta c_2 = 10^{-4}$ every 5000 time steps for $N = 63$, $\nu = 0.1$ and $\eta = 0.67$. Oscillators are colored by the clusters to which they belong in the cluster-splitting cascade. Initially, there is a cluster of 32, reaching a single blue maximum and a cluster of 31 reaching a single red maximum. At $c_2 \approx -0.65$, the latter splits up into a cluster of 16, shown in black, and a cluster of 15, shown in red, that both are period-2. At $c_2 \approx -0.637$, the cluster of 15 splits up into a cluster of eight (yellow) and a cluster of seven (red). At $c_2 \approx -0.7365$, the cluster of seven splits up into a cluster of four (purple) and a cluster of three (red). **b**, Cluster sizes at each value of c_2 during the c_2-incremented simulation in **a**. Calculations are based on the cross correlations of trajectories (in the non-rotating frame) over the last 800 time steps of simulation at each c_2 value and a threshold of $\varepsilon = 10^{-8}$. See Methods section.
